# Etiology, clinical findings and laboratory parameters in neonates with acute bacterial meningitis

**Published:** 2020-04

**Authors:** Hassan Boskabadi, Elahe Heidari, Maryam Zakerihamidi

**Affiliations:** 1 Department of Pediatrics, School of Medicine, Mashhad University of Medical Sciences, Mashhad, Iran; 2 Department of Midwifery, Faculty of Medical Sciences, Tonekabon Branch, Islamic Azad University, Tonekabon, Iran

**Keywords:** Meningitis, Newborn, *Klebsiella pneumoniae*, *Enterobacter aerogenes*, Laboratory diagnosis

## Abstract

**Background and Objectives:**

Neonatal meningitis is one of the most important and serious neonatal infections with a high mortality and morbidity rate. The present study aimed to investigate the causes, clinical signs, laboratory parameters and mortality rates in newborns with bacterial meningitis.

**Materials and Methods:**

This cross-sectional study was performed on 468 neonates aged 2-28 days admitted to NICU in Ghaem Hospital Mashhad, Iran by available sampling method during 2009–2018. Meningitis was confirmed according to positive results of CSF culture and clinical feature. By using researcher-made questionnaire, neonate’s individual data including cardiopulmonary resuscitation, the Apgar score of the first and fifth minutes, gestational age, birth weight, clinical symptoms and laboratory data such as ESR, WBC and positive culture of CSF were studied.

**Results:**

Among 468 newborn suspected to infection, lumbar Puncture (LP) was performed for 233 cases (50%). Of 233 neonates, 148 neonates (63.5%) had negative results for CSF culture and 85 cases (36.5%) had positive CSF culture. 94% of cases with meningitis were born premature. Blood culture had positive results in 80% of infants with late-onset meningitis and negative in 20%. The most common clinical findings were respiratory symptoms (94%). *Klebsiella pneumoniae* and *Entrobacter aerugenes* were the most common microorganisms of meningitis. Gestational disorders were observed in 55.3% of newborns with meningitis. C-Reactive Protein (CRP) of neonates with meningitis was twice higher than normal cases, and leukocytes and proteins in the CSF in neonates with meningitis were higher than healthy ones. Finally, 36% of neonates with meningitis died in our study. For analyzing the relationships between variables, independent t-test was used after controlling the normality, and Chi-square was used for analyzing the relationship of variables with nominal scale.

**Conclusion:**

The most common pathogens of meningitis were *Klebsiella pneumoniae* and *Enterobacter aerogenes*. Respiratory symptoms were the most common clinical signs, and laboratory symptoms included increased CRP, increased leukocytes and proteins in CSF.

## INTRODUCTION

Neonatal infections are serious diseases with high mortality, and are the most important concerns of neonatal care professionals during the admission of each infant due to non-specific symptoms as well as lack of an early and accurate diagnostic test. Neonatal meningitis is one of the most important and dangerous infections (1) and a major cause of morbidity and mortality worldwide (2). Despite development in neonatal intensive care, neonatal meningitis is still considered a lethal disease (3). Neonatal meningitis is responsible for up to 10–15% of mortality (4), and 25–50% of deafness, blindness, cerebral palsy, seizure, hydrocephalus, or cognitive impairment of surviving infants (2). Bacterial meningitis can cause acute complications such as brain parenchymal vasculitis, ventriculitis and systemic complications such as pneumonia and septic shock (5).

Knowledge about causative pathogens of meningitis helps for prompt diagnosis and treatment. In developing countries such as India, despite important advances in treatment, mortality rates of this disease is still 16–34% (6, 7).

Unfortunately, mortality and morbidity rate of meningitis and its expenses have not been determined in Iran. In the United States, meningitis accounts for about 72,000 hospitalized cases per year and costs 1.2 billion dollars annually (8). Surveys in 2010 found that meningitis caused 422,900 deaths and 262,800 cases of neurological disorders (9). The prevalence of meningitis in Asia is 0.48–2.4 in 1,000 live births. Although a slight decrease in incidence of meningitis has been occurred between 2003 and 2013, nothing has been observed about survival rate of patients and reduction in long-term morbidity in Iran over several decades (10). Therefore, many efforts have been made to improve the treatment and prevention for bacterial meningitis (11). Neonatal cerebrovascular system is very vulnerable compared to adults, and it is very sensitive to drugs, toxins and pathological conditions. Also after birth, loss of body defense occurs due to separation from the placenta. Therefore, brain is prone to damage and ultimately nerve disorders (12).

Acute meningitis can be bacterial, viral, fungal, or parasitic. Bacterial pathogens can cross the blood brain barrier leading to cell damage. Many of neurons produce the cytokines, chemokines, and other pro-inflammatory molecules in response to bacterial stimulation, and activate polymorphonuclear leukocytes and result in oxidative stress (2). Pathogens responsible for acute bacterial meningitis vary according to the studied population, patients’ age, and location of study (13). Clinical diagnosis of meningitis during neonatal period is very difficult because clinical manifestations of bacterial meningitis are often non-specific, and lumbar puncture is often postponed in neonates with unstable clinical status (14).

On the other hand, clinical symptoms of patients with meningitis vary according to the type of pathogen, age, gender, and duration of disease (15, 16). Clinical symptoms include hyperthermia, poor feeding, vomiting, lethargy and restlessness, and clinical signs include bulging of fontanel, fever, drowsiness, apnea and seizure (17). Diagnosis of acute bacterial meningitis is based on the combination of clinical signs and symptoms, laboratory data and inflammatory response in CSF, and detecting specific bacterial organism (by Gram staining, culture, antigen and molecular identification) (18). CSF parameters are especially helpful in cases where diagnosis has not been confirmed, or in cases of bacterial meningitis without bacteremia (14). Laboratory parameters of acute bacterial meningitis include neutrophilic pleocytosis with increased protein and decreased glucose in CSF (7). Therefore, when patients are suspected to be septic in this age group, they should be admitted, and CSF should be obtained. Subsequently, they are treated with antibiotics empirically until confirmation of result of CSF culture (19).

Apgar is a quick test performed on a baby at 1 and 5 minutes after birth. The 1-minute score determines how well the baby tolerated the birthing process. The 5-minute score tells the health care provider how well the baby is doing outside the mother’s womb. The Apgar test is done by a doctor, midwife, or nurse. The provider examines the baby’s: Breathing effort, Heart rate, Muscle tone, Reflexes, Skin color (20).

Knowledge about common pathogens of meningitis, clinical symptoms, laboratory parameters, risk factors, complications and cost estimation is necessary in patient management. Despite the fact, there is not enough information about the epidemiology of neonatal bacterial meningitis in Iran, and acute bacterial meningitis is considered as a public health risk with morbidity, mortality and high financial burden for health care providers (15). This study was aimed to investigate the causes, clinical signs, laboratory parameters and mortality rates in newborns with bacterial meningitis.

## MATERIALS AND METHODS

This cross-sectional study was performed on 468 neonates aged of 2–28 days who were admitted to the Neonatal Intensive Care Unit (NICU) of Ghaem Hospital in Mashhad, Iran with available sampling method from 2009 to 2018. Neonates whose parents did not give consent for LP, and those with cytological CSF parameters suspected to meningitis but had negative results for CSF culture were excluded. Of 234 neonates (50%), CSF sample was obtained for smear, culture and cell, sugar and protein analysis. Based on the results of positive CSF culture and clinical signs, meningitis was confirmed, and infants with confirmed meningitis were included in this study. Neonates with negative results in culture were defined as control group. This study was approved by the research committee of Mashhad university of Medical sciences (IR.MUMS.fm.REC.1396.115). A written informed consent was obtained from patient’s parents before entering the study.

Using a researcher-made questionnaire, personal information were assessed including cardiopulmonary resuscitation, the Apgar scores in the first and fifth minutes, gestational age, birth weight, admission weight, head circumference, height, sex, clinical symptoms, reason for admission, duration of mechanical ventilation, duration of oxygen therapy, Intraventricular hemorrhage (IVH) in ultrasound, final diagnosis, causes of death, personal data such as maternal age, parity, gestational problems, delivery, and laboratory data including microorganisms of CSF positive culture, Erythrocyte Sedimentation Rate (ESR), Urea, Cr, Na, K, total bilirubin, direct bilirubin, Prothrombin Time (PT), Partial Thromboplastin Time (PTT) and WBC; neutrophil percentage; lymphocyte percentage; platelets at time of infection, granulated RBC, C-Reactive Protein (CRP), protein; sugar; leukocyte; neutrophil; lymphocyte and RBC in CSF. More than 30 leukocytes per mm 2 of CSF with neutrophils priority, increased CSF more than 150 mg/dL, decreased glucose in CSF (less than 70% of blood sugar level, concurrently), and identification of bacteria in microscopic examination and culture of cerebrospinal fluid were considered as bacterial meningitis (21). Diagnosis of meningitis was confirmed by positive results for cultures, clinical symptoms with or without abnormal results of CSF (increasing the number of polymorphonuclear leukocytes and high protein concentration over than 120 mg/dL) (21).

Data analyzed using SPSS version 24 (SPSS Inc., Chicago, Ill, USA). Data normal distribution was checked by Kolmogorov-Smirnov test. For normal data, independent t-test and Chi-square were used for quantitative and qualitative variables, respectively. P<0.05 was considered as statistically significant.

## RESULTS

Of 468 infants suspected to infection, CSF was examined in 234 infants (50%). Of 234 neonates, 148 (63.5%) had negative results for CSF culture and 85 (36.5%) had meningitis. In evaluation of neonates with meningitis, 49 neonates (57.65%) were female and 36 neonates (42.35%) male. Twenty neonates (23.53%) experienced cardiopulmonary resuscitation at birth. Totally, 54 newborns (63.5%) were born by normal vaginal delivery and 31 neonates (36.5%) by cesarean section. Gestational problems included chorioamnionitis (8 cases, 9.41%), preeclampsia (4 cases, 4.71%), eclampsia (1 case, 1.18%), placental abruption (2 cases, 2.35%), hypertension (6 cases, 7.06%), gestational diabetes (1 case, 1.18%), uterine adhesion (2 cases, 2.35%), hypothyroidism (6 cases, 7.06%), epilepsy (1 case, 1.18%), and Premature Rupture of Membranes (PROM) (16 cases, 18.82%). The Mean of variables of the first minute Apgar score was 6.86 ± 2.19, gestational age 30.53 ± 2.68 weeks, and birth weight 1314.88 ± 413.01 grams. Other maternal and neonatal variables are presented in Table 1.

**Table 1.** Mean of maternal and neonatal parameters in newborns with meningitis

**Variables**	**Mean ± SD**
Maternal age (year)	4.99 ± 29.93
Parity	1.26 ± 2.66
First minute Apgar score	2.19 ± 6.86
Fifth minute Apgar score	1.67 ± 7.61
Gestational age (week)	2.68 ± 30.53
Birth weight (gram)	413.01 ± 1314.88
Admission weight (gram)	533.21 ± 1343.25
Head circumference (cm)	4.12 ± 28.14
Height (cm)	3.47 ± 38
Mechanical ventilation duration (hour)	16.37 ± 16.60
Oxygen therapy duration (day)	6.17 ± 6.30
ESR (mm/hr)	14.05 ± 11.28
Urea (mg/dL)	12.23 ± 54.95
Cr (mg/dL)	0.86 ± 0.35
Na (mEq/L)	7.23 ± 141.68
K (mEq/L)	0.57 ± 4.95
Bilirubin (mg/dL)	3.26 ± 6.81
Direct bilirubin (mg/dL)	0.58 ± 0.66
PT (second)	7.75 ± 19.50
PTT(second)	34.71 ± 65.75
WBC at time of infection	11.01 ± 13.49
Percentage of neutrophils at infection	16.24 ± 50.84
time	
Percentage of lymphocytes at the	16.40 ± 42.35
time of infection	
Platelet during infection	126.17 ± 175.80
Nucleated red blood cells	14.78 ± 6.07
(NRBC/100 WBC)	

**Fig. 1.**
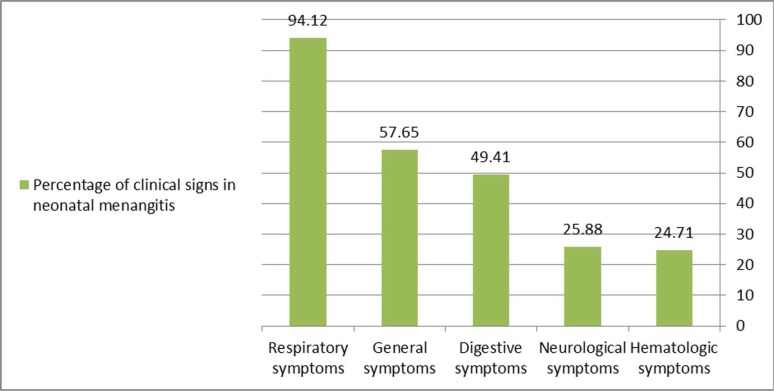
Clinical signs of neonates with meningitis

The reasons for hospitalization were as 29 neonates (34.12%) with low Apgar score, 48 (56.47%) due to respiratory distress and prematurity, 3 (3.53%) with congenital anomalies, 3 (3.53%) with apnea and 2 (2.36%) due to irritability. Clinical signs of infants (general, neurologic, cardiac, digestive, hematologic and respiratory) are summarized in Table 2 and Fig. 1.

**Table 2.** Clinical findings of neonates with meningitis

**Clinical signs of neonates with meningitis**	
General symptoms 49 neonates (57.65%)	Cyanosis (22 cases)
	Metabolic acidosis (10 cases)
	Bad General status (8 cases)
	Lethargy (5 cases)
	Poor feeding (2 cases)
	Poor peripheral perfusion (2 cases)
Neurological symptoms 22 neonates (25.88%)	Seizure (10 cases)
	CNS hemorrhage (3 cases)
	Hydrocephalus (2 cases)
	Myelomeningocele (2 cases)
	Hypertonia (2 cases)
	Hypotonic (2 cases)
	Bulging of fontanel (1 case)
Respiratory symptoms 80 newborns (94.12%)	Low SPO2 (34 cases)
	Mechanical ventilation (33 cases)
	Tachypnea (27 cases)
	CPAP (25 cases)
	Granting (16 cases)
	Retraction (2 cases)
	Nasal flaring (1 case)
Digestive symptoms 42 newborns (49.41%)	Abdominal distention (22 cases)
	Vomiting (16 cases)
	Feeding intolerance (3 cases)
Hematologic symptoms 21 (24.71%)	Thrombocytopenia (13 cases)
	DIC (7 cases)
	Anemia (1 case)

The final diagnosis of neonates were as follows: prematurity (41 cases), asphyxia (33 cases), myelomeningocele (1 case), hydrocephalus (1 case), respiratory problems (8 cases), Respiratory Distress Syndrome (RDS) (30 cases), pulmonary hemorrhage (4 cases), pneumonia (2 cases), Bronchopulmonary dysplasia (BPD) (1 case), Necrotizing enterocolitis (NEC) (1 case), congenital anomalies (4 cases) and Conjunctivitis (3 cases). Microorganisms that were detected in positive CSF culture were as follows; *Klebsiella pneumoniae* 34 cases (48.6%), *Enterobacter aerogenes* 10 cases (14.3%), *Streptococcus* group D 8 cases (11.4%), *Acinetobacter* 4 cases (5.7%). *Escherichia coli* 3 cases (4.3%), *Staphylococcus epidermidis* 3 cases (4.3%), *Enterobacter* 3 cases (4.3%), *Pseudomonas aeruginosa* 2 cases (2.9%), *Staphylococcus saprophyticus* 1 case (1.4%), *Enterococcus faecalis* 1 case (1.4%), *Candida albicans* 1 case (1.4%). Comparison of mean neonatal variables in cases with and without meningitis is shown in Table 3.

**Table 3.** Comparison of mean neonatal variables in cases with and without meningitis

**Groups**	**Neonates without meningitis**	**Neonates with meningitis**	**Significant level**
**variables**	**148 newborns (63.5%)**	**85 neonates (36.5%)**	**(T-Test)**
First minute Apgar score	6.93 ± 2.13	5.86 ± 2.19	0.822
Fifth minute Apgar score	7.54 ± 1.42	7.61 ± 1.67	0.748
Gestational age (week)	30.29 ± 3.17	30.53 ± 2.68	0.554
Weight (gram)	1321.05 ± 496.09	1314.88 ± 413.1	0.926
First ESR (mm/hr)	7.27 ± 14. 39	11.28 ± 14.05	0.338
Urea (mg/dL)	42.18 ± 21.76	54.95 ± 12.33	0.002
Bilirubin	7.43 ± 2.69	6.81 ± 3.26	0.445
Direct bilirubin	0.45 ± 0.21	0.66 ± 0.58	0.051
CRP	23.00 ± 21.05	41.43 ± 25.45	0.003
Hematocrit	41.13 ± 6.24	40.79 ± 7.55	0.788
WBC at the infection time	13.61 ± 11.98	13.49 ± 11.01	0.962
Neutrophil at infection time	51.54 ± 15.62	50.84 ± 16.24	0.852
Lymphocyte at infection time	38.08 ± 17.15	42.35 ± 16.40	0.365
CSF protein	88.23 ± 44.27	140.85 ± 152.70	0.000
CSF sugar	63.93 ± 47.31	64.68 ± 27.63	0.976
Leukocyte	69.2 ± 5.93	522.80 ± 849.40	0.000
CSF			
CSF Neutrophil	78.40 ± 9.94	85.33 ± 21.93	0.091
CSF lymphocyte	14.66 ± 10.98	14.66 ± 21.93	0.245

* The values are based on mean ± standard deviation

## DISCUSSION

Based on the results of present study, clinical signs of neonatal meningitis are very non-specific and the common microorganisms of neonatal meningitis are Gram-negative bacteria and mortality rate is very high. In this study 85 neonates (36.5%) had positive results for CSF culture, and about 1.5% of infants suspected for sepsis detected to have meningitis in our departments. In one study, positive result for CSF culture was reported in 36.88% (7). In another study, the prevalence of neonatal meningitis was 1% (22).

In this study, gestational problems were seen in 55.3% of cases with meningitis, and the most prevalent were PROM (19%), chorioamnionitis (9%), and hypertension (7%). In a study, the probability of bacterial meningitis in neonates with PROM was 5.2% (23). Based on the results of a systematic review, one of the main factors in the occurrence of PROM is infection (mostly bacterial infection) that stimulates the release of pro-inflammatory cytokines from decidua and amniotic membranes. Therefore, many bioactive materials, such as prostaglandins and metalloprotease are released. So prostaglandins stimulate the uterine contractions, and metalloprotease cause the cervical softness and eventually tearing the membranes occurs (24). Maternal infection leading to premature rupture of membrane may be transmitted to the fetus via placenta or vaginal canal and result in sepsis and neonatal meningitis. According to the results of a study, the role of maternal genitourinary infections is obvious in neonatal meningitis. So that, 45.4% of neonates with meningitis had mothers with Urinary Tract Infection (UTI) (25). The results of one study showed that the risk factors for prenatal neonatal meningitis include PROM, maternal vaginitis and asymptomatic bacteriuria (26). Insufficient care or lack of care during pregnancy increases the chance of mother-to-baby infection transmission.

In assessment of neonates with meningitis, 49 neonates (57.65%) were female and 36 neonates (42.35%) male and gender did not show any significant association with meningitis, and mortality of female neonates was more than males. This finding was similar to the results reported by Zamani et al. (27). In another study, in contrast to our findings, mortality rate of meningitis was higher in male neonates than in females (16). The other study, 68.6% of infants with proven meningitis were male (28).

In the present study, 94% of cases with meningitis were born premature and the mean gestational age was 30.53 ± 2.68 weeks. In many studies, 28–43% of neonates with meningitis were premature (27–29). In a prospective study on 144 newborns with meningitis, mortality rate in preterm infants was more than twice rather than term neonates (26% vs. 10%) (4). Meningitis with late onset is commonly seen in premature neonates and has a direct relation with reduced gestational age and birth weight (30). Knowing that most maternal immunoglobulin do not cross the placenta before the 32 ^nd^ week of gestation, very premature neonates are at high risk of infection, including meningitis (31).

Blood cultures had positive results in 80% of newborns with late onset meningitis and negative in 20% of cases. In case of early onset meningitis, blood culture had positive results in 21% and negative in 78% of patients. In the Nel’s study (2000), 88% of cases with neonatal meningitis showed positive CSF culture (32). Neonatal meningitis often occurs following bacteremia and, in few cases, it occurs due to adjacent transmission or neurologic anatomical defect. Usually, as our study showed, the incidence of meningitis in late sepsis is greater than early sepsis.

The most common clinical symptoms of meningitis among neonates in the recent study was respiratory symptoms (94%) which may be related to underlying illness of patient, or respiratory problems of newborns lead to frequent medical interventions that makes patient vulnerable to infection. Sometimes, general condition of neonates with meningitis leads to apnea which necessitates mechanical ventilation. In Khalesi et al. (2014), 30% of infants with neonatal meningitis had tachypnea (26).

The second group of common symptoms was non-specific and general. In one study, poor feeding, lethargy, hyperthermia, followed by seizure, hypothermia and vomiting were among the most common symptoms of neonates with bacterial meningitis (27). In another study, poor feeding was the most common (60%) clinical sign among neonates with neonatal meningitis (26).

The third group of clinical symptoms was gastrointestinal findings. Tan et al. (2015) reported vomiting in 10.8% of patients with neonatal meningitis, which was one of the predictors of poor prognosis in neonates (33).

Neurological symptoms were observed in 24% of neonates with meningitis. In the study of Devi et al. (2017) seizure and lethargy were the most common clinical symptoms in neonates with meningitis (28). In Khalesi et al. (2014), seizure was prevalent as 55% in neonatal meningitis (26). Neonates are susceptible to meningitis and its related neurological complications. Despite progress in neonatal care, neonatal bacterial meningitis leads to neurological complications in 20–58% of neonates with meningitis (34).

The last group of clinical findings was hematological data. Thrombocytopenia was present in 40% of neonates in the present study. In one study, thrombocytopenia was found in 13.3% of neonates with meningitis (27). In another retrospective study, thrombocytopenia was an important predictor for neonatal meningitis complications (35). In a study, anemia with hemoglobin less than 14.5 g/dL was observed in 151 (65%) neonates with meningitis (33).

The most common microorganisms in patients with positive results for CSF culture were *Klebsiella pneumoniae* (49%), *Enterobacter aeruginosa* (14%), *Streptococcus* group D (11%) and *Acinetobacter baumannii* (6%). Early onset bacterial meningitis is caused by pathogens colonized in maternal vagina or at delivery ward (36). In one study, *Escherichia coli* (5%) and *Klebsiella* (4%) were the most common Gram-negative pathogens for late- onset infections (37). The most common microorganisms in Nel’s study were the group B streptococci, *K. pneumoniae* and *E. coli* (32) .

In one study, *K. pneumoniae*, Coagulase negative staphylococci, and *Enterococcus faecalis* were the most common bacterial pathogens (28). In the study of Samiee Rad et al. (2012), the most common pathogens were *E. coli, K. pneumoniae, Enterobacter, Pseudomonas aeruginosa* and *Staphylococcus aureus* (22). Several mechanisms have been described for neonatal meningitis, but primary septicemia and then the secondary distribution in cerebrospinal fluid is the most common underlying issue (38). This is the reason why neonatal sepsis is present in approximately 75% of cases with neonatal meningitis, concurrently (39).

CRP in neonates with meningitis is reported to be twice more than those without meningitis. CRP is an acute phase reactant that rises 6–18 hours after infection. Its sensitivity in diagnosis of neonatal infections is about 30% and the specificity is about 60% (40). In another study, 72% of neonates with meningitis showed CRP level higher than 8 mg/Dl (33). The result of a study showed that CRP level of cerebrospinal fluid in neonates with purulent meningitis was higher than aseptic meningitis. Therefore it can be concluded that CRP can be considered as a sensitive reactive agent for differentiation of bacterial and aseptic meningitis (41).

In our study, leukocytes in CSF of neonates with meningitis was significantly higher. To prove the neonatal meningitis based on CSF culture, WBC count more than 21 cells/mm ^3^ is possessed with a sensitivity of 97% and specificity of 81% (38, 42). The results of a study on 1064 neonates under 28 days age without bacterial meningitis showed that the median of CSF was 3 cells (WBC) per milliliter, and its 95th percentile was 19 cells per milliliter (43). In another study, the number of WBCs showed statistically significant association with neonatal bacterial meningitis (27). In our study, the protein content of CSF was significantly higher in neonates with meningitis. The results of Devi’s et al. (2017) study showed that increased CSF protein occurs in 12% and decreased CSF glucose in 7.5% of newborns with bacterial meningitis (28). In one study, increased level of CSF protein was one of the poor prognostic factors in neonates with meningitis (44).

In another study, the level of glucose and CSF protein were significantly associated with neonatal bacterial meningitis (27). Garges et al. (2006) reported the normal CSF parameters in neonatal meningitis (42). In Nel’s study, increased CSF protein was associated with an increased risk of mortality (32).

Totally, 36% of newborns with meningitis died in our study. In another study, 34% of patients with neonatal meningitis died, and most death occurred during the first 72 hours after admission (32). In Radouani et al. (2014), study neonatal bacterial meningitis was associated with a mortality rate of over than 10% (45).

The main limitation of our study was the control group, that LP was performed for them and showed clinical and laboratory symptoms of infection rather than meningitis.

## CONCLUSION

According to the results of the present study, the causes of neonatal meningitis in our ward included *K. pneumoniae* (49%), *E. aeruginosa* (14%), *Streptococcus* group D (11%), and *Acinetobacter* (6%). Clinical signs of neonatal meningitis were non-specific including respiratory symptoms (94%), general symptoms (58%), gastrointestinal symptoms (49%), neurological symptoms (26%), and hematological symptoms (25%). In this study, 94% of cases with meningitis were born as premature. Blood cultures were positive in 80% of cases with late meningitis and in 22% in early onset meningitis, and negative in 20% of cases with late meningitis, and in 78% of early meningitis. In case of early meningitis, blood cultures had positive results in 21% and negative in 78%. Increased leukocytes and protein of CSF and also CRP and Urea were laboratory findings of meningitis. Finally, 36% of neonates with meningitis who have laboratory symptoms died in our study.
